# Repeated Bout Effect of Two Resistance Training Bouts on Bowling-Specific Performance in Male Cricketers

**DOI:** 10.3390/sports10090126

**Published:** 2022-08-24

**Authors:** Drew C. Harrison, Kenji Doma, Anthony S. Leicht, Teneale A. McGuckin, Carl T. Woods, Jonathan D. Connor

**Affiliations:** 1 Sport and Exercise Science, James Cook University, Townsville, QLD 4811, Australia; 2 Australian Institute of Tropical Health & Medicine, James Cook University, Townsville, QLD 4811, Australia; 3 Institute for Health & Sport, Victoria University, Melbourne, VIC 3011, Australia

**Keywords:** resistance training, muscle damage, delayed-onset muscle soreness, repeated bout effect, cricket

## Abstract

To examine the repeated bout effect (RBE) following two identical resistance bouts and its effect on bowling-specific performance in male cricketers. Male cricket pace bowlers (N = 10), who had not undertaken resistance exercises in the past six months, were invited to complete a familiarisation and resistance maximum testing, before participating in the study protocol. The study protocol involved the collection of muscle damage markers, a battery of anaerobic (jump and sprint), and a bowling-specific performance test at baseline, followed by a resistance training bout, and a retest of physical and bowling-specific performance at 24 h (T24) and 48 h (T48) post-training. The study protocol was repeated 7–10 days thereafter. Indirect markers of muscle damage were lower (creatine kinase: 318.7 ± 164.3 U·L^−1^; muscle soreness: 3 ± 1), whilst drop jump was improved (~47.5 ± 8.1 cm) following the second resistance training bout when compared to the first resistance training bout (creatine kinase: 550.9 ± 242.3 U·L^−1^; muscle soreness: 4 ± 2; drop jump: ~43.0 ± 9.7 cm). However, sport-specific performance via bowling speed declined (Bout 1: −2.55 ± 3.43%; Bout 2: 2.67 ± 2.41%) whilst run-up time increased (2.34 ± 3.61%; Bout 2: 3.84 ± 4.06%) after each bout of resistance training. Findings suggest that while an initial resistance training bout reduced muscle damage indicators and improved drop jump performance following a second resistance training bout, this RBE trend was not observed for bowling-specific performance. It was suggested that pace bowlers with limited exposure to resistance training should minimise bowling-specific practice for 1–2 days following the initial bouts of their resistance training program.

## 1. Introduction

Cricket is a multidimensional sport involving a variety of complex motor skills (i.e., interceptive, striking, far aiming tasks) [1]. Bowling is an example of a far aiming task and is influenced by changes in technical, physical, or morphological characteristics of the individual [2]. Bowlers can manipulate variables such as ball trajectory and delivery length, although, a key aspect of bowling is ball speed, particularly for pace bowlers [3].

Pace bowling requires repeated, near maximal high-intensity efforts, with ball speeds approximating 80–160 km/h [4]. Resistance training (RT) has been purported as an effective conditioning approach to optimise performance for pace bowlers by increasing muscular strength and power [5]. However, strenuous RT could result in significant levels of muscle damage, particularly for individuals unaccustomed to exercise [6]. Known as exercise-induced muscle damage (EIMD), the characteristics of this acute trauma include muscle soreness, stiffness, swelling, decreased muscular contractility, and elevated secretion of creatine kinase (CK) [7,8,9]. Due largely to the disruption of the intricate myofibril structure [10], EIMD symptoms peak 24–48 h post-exercise, with some studies reporting residual effects up to 7–14 days in resistance-untrained individuals [11,12,13]. Further, EIMD compromises muscle function required during basic motor skills, including jumping [14], sub-maximal running [15,16], and sprint performance [17].

To date, the number of studies that have reported the attenuation of basic motor skills (e.g., jumping and running) during periods of EIMD has been extensive, while the investigation of EIMD on sport-specific performance measures involving speed and accuracy has been limited. In a recent study [18], a RT bout consisting of multiple upper and lower body exercises impaired ball speed and bowling accuracy during a bowling performance protocol for up to 48-h post-exercise in adolescent male, cricket fast bowlers. In addition, vertical jump, sprint performance, and run-up speed during the bowling performance protocol were also compromised. Doma, Leicht, Woods, Harrison, McGuckin and Connor [18] purported that the impairment in muscular contractility caused by EIMD may perturb run-up speed and mechanics during a bowling action, thereby compromising ball speed and accuracy. However, Doma, Leicht, Woods, Harrison, McGuckin and Connor [18] only examined the acute effect of one RT bout on bowling performance measures, despite evidence indicating a reduction in EIMD symptoms following multiple RT bouts, known as the repeated bout effect (RBE) [11,19]. Although the underlying mechanisms encompassing RBE are not yet completely understood, the acute adaptation following a second bout of RT is thought to be a result of improved efficiency in neural, cellular, and mechanical processes involved in RT [19]. Several studies have reported lower levels of CK and DOMS, with faster recovery of muscular function, vertical jump, sub-maximal running, and sprint performance measures following the second bout of RT [15,20,21]. Consequently, the recovery dynamics are accelerated in response to multiple RT bouts, thereby minimising the detrimental impact of EIMD on subsequent physical performance.

According to the above findings, athletes should avoid training that involves jumping and running actions following the first RT bout during the initial period of a resistance training program (e.g., youth athletes commencing a resistance training program, or those following a training hiatus from an injury). However, the impact that EIMD has on physical performance also depends on the type of motor task with more complex tasks less impacted, possibly due to greater compensatory movement patterns to maintain performance [16]. For example, Doma, Connor, Gahreman, Boullosa, Ahtiainen and Nagata [22] recently showed that the initial RT bout returning from the off-season impaired change-of-direction speed and spiking speed for up to 24 h post-exercise in female volleyball players, although their accuracy was improved. These findings suggest a speed-accuracy trade-off, with athletes possibly adjusting their complex motor pattern to maintain, or enhance, performance whilst accounting for impaired muscular function. Contrarily, Doma, Leicht, Woods, Harrison, McGuckin and Connor [18] showed attenuation in both speed and accuracy during a bowling task for up to 48 h following a RT bout. The discrepancy in findings between spiking [22] and bowling performance [18] suggests that the type of sport-specific task may influence the effect of EIMD on performance outcomes.

The evidence, to date, indicates a RBE trend for basic motor tasks, such as jumping and running [15,16,21]. However, these activities do not translate to the complexity of a sport-specific skill that involves speed and accuracy, such as a bowling action in cricket. Therefore, the purpose of this study was to investigate the emergence of a RBE following two consecutive bouts of RT on cricket bowling-specific performance measures. It was hypothesised that following the second bout, the RBE would attenuate the presence of EIMD, reducing the concomitant decline in indirect markers of muscle damage and allowing athletes to adjust their cricket-bowling action.

## 2. Methodology

### 2.1. Participants

Ten skilled male cricket pace bowlers (mean age 18 ± 2 yrs; height 1.78 ± 5.3 m; weight 71.2 ± 4.2 kg; BMI 23.2 ± 5.0 kg/m^2^) were invited to participate. Participants were excluded if previous exposure to RT was within the last 6 months, or if they experienced any current acute or chronic illness, disease, injury, or consumed medication that would affect the subsequent testing protocols. Participants were also requested to refrain from strenuous activity for at least 48 h prior to testing other than the study protocol and maintain dietary habits during the study. Written informed consent was obtained from all participants and guardians prior to commencing the study. Ethical approval was obtained from the university’s Human Research Ethics Committee (H8066). Based on *a priori* sample size calculations and previous studies (Doma et al. [18]; Doma et al. [20]), 10 participants were sufficient to detect a significant change in variables with power set at 80% and alpha level of 0.05.

### 2.2. Research Design

The study was conducted as part of a larger research project using similar protocols and outcome measures [18]. The study was conducted over a four-week period (Table 1). The first week involved a familiarisation session and repetition maximum (RM) assessment session. The familiarisation session was used to acquaint the participant with the bowling-specific performance test, anaerobic performance assessments, and the collection of indirect muscle damage markers. The RM assessment session was used to determine 1 RM of incline leg press (AMF-656, APT Sports, Adelaide, SA, Australia), single leg horizontal leg press (NS4000, Nautilus, Winnipeg, MB, Canada) chest press (NS4000, Nautilus, Winnipeg, MB, Canada), and 6 RM for seated row (NS4000, Nautilus, Winnipeg, MB, Canada) and triceps extension (NS4000, Nautilus, Winnipeg, MB, Canada).

During the second week, a full body RT session was completed, with indirect muscle damage markers, anaerobic performance, and bowling-specific performance measures collected prior to (Tbase) and 24 (T24) and 48 h (T48) after the RT session. The study protocol was repeated 7–10 days thereafter.

### 2.3. Repetition Maximum Assessment

Prior to the 1 RM and 6 RM assessments, participants completed a standardised warm-up consisting of a walk on a treadmill for three minutes at 6–8 km/h, followed by a mobility warm-up of the lower (i.e., glute bridges, leg abduction, and leg adduction) and upper (i.e., external rotation, internal rotation, and Palloff press) extremities. Participants completed two warm-up sets of ten and six repetitions for incline leg press, chest press, and seated row exercises at 80% of their body mass for the lower extremity and 40% of the 6 RM for upper extremity. A two-minute rest was allowed in between each warm-up set. After the warm-up sets, a standard repetition maximum assessment was conducted following the National Strength and Conditioning Association guidelines (Baechle & Earle, 2008). Final load was achieved within three to five attempts and each attempt was separated by five minutes of rest. Participants were instructed to refrain from any other strenuous exercise or dietary supplementation outside of their regular diet throughout this study.

### 2.4. Indirect Muscle Damage Markers

The indirect markers of muscle damage included CK and delayed-onset muscle soreness (DOMS) in the lower body. The DOMS measures consisted of participants reporting their level of soreness whilst sitting down on the ground (SitSore), their thigh soreness whilst isometrically contracting their right knee extensors with their knee in an extended position (ThighSore), and their hamstring soreness whilst isometrically contracting their hamstrings with their right knee in approximately 90° flexion (HamSore). Participant DOMS was measured using a 10-point visual analogue scale, where one was denoted as “no soreness” and ten as “very, very sore” [16]. A capillary blood sample of 30 µL was collected via finger prick from the participant’s non-bowling hand to assess CK. The blood sample was immediately syringed onto a test strip before it was assessed via a colorimetric assay (Reflotron, Boehringer Mannheim, Mannheim, Germany). The intra-assay coefficient of variation for CK within our laboratory was 7.2%.

### 2.5. Anaerobic Performance and Sprint Tests

Sequential assessments of counter-movement jump (CMJ), DJ, and a fifteen-metre sprint were conducted to determine measures of anaerobic power. For the DJ assessment, participants completed three maximal jumps from a thirty-centimetre box and tasked to reach as high as possible on the vertical jump apparatus (YardStick, Swift Performance, BRISBANE, QLD, Australia), with thirty seconds of passive recovery between each trial. The CMJ protocol was completed similarly, although commenced from the ground. Instructions were provided to participants and rest time between jumps (30 s) was standardised across all participants. Finally, participants completed three, 15 m sprints with three minutes of rest between each trial, with timing gates positioned at 10 m and 15 m. The distance of 15 m was selected for the sprint protocol to replicate common distances covered by a pace bowler during the run-up [23]. The timing gates were also positioned at 10 m to report on time-to-completion at varying distances. All participants started each sprint 0.5 m behind the first timing gate (Speedlight, Swift Performance, Brisbane, QLD, Australia) with the two other timing gates placed at five-metre intervals. The best sprint time was recorded and used for later analysis.

### 2.6. Bowling-Specific Performance Test

The bowling-specific performance test was identical to that employed previously (refer to study by Doma, Leicht, Woods, Harrison, McGuckin and Connor [18] for specific detail of the protocol). In summary, participants bowled a total of 18 deliveries, which were separated into 3 groups (i.e., three overs) of 6 deliveries. Ball speed was recorded during each delivery using a radar speed camera (Pocket Radar, Ball Coach, Santa Rosa, CA, USA) adjacent to the bowler’s preferred side. Average and peak ball speed was calculated as well as the best values across the six deliveries from the first, second, and third overs.

Bowling accuracy was monitored through visual inspection by using a target mat placed on the cricket pitch. The dimensions of the target were 1 m × 4 m and placed longitudinally along the wicket 2 m in front of the stumps. This position is representative of a ‘good length’ delivery and is a common target zone during training and match circumstances [24]. The target was separated horizontally, from left to right, into red-, orange-, and yellow-coloured zones. Participants were instructed to bowl at the red-, orange-, and yellow-coloured zones for overs one, two, and three, respectively.

The bowling accuracy was calculated based on a standardised scoring system with three points given if the ball landed within the correct coloured zone in the ‘good ball length’, two points given if the ball landed in the ‘good ball length’ but not the correct coloured zone, one point given if the ball landed in the correct coloured zone line, and zero scores given if the ball missed the entire target area. Bowling accuracy was calculated as a percentage of the best score possible (i.e., 3 overs × 6 bowls × best score of 3 per bowl = 54) across the three overs (Accuracy [%] = total score/54 × 100).

The running speed during each bowling delivery was measured using an electronic timing gate system (Speedlight, Swift Performance, Australia). Like the bowling speed measures, the average and peak run-up completion times were calculated as the average and best of the six bowls for the first, second, and third overs.

### 2.7. Resistance Training Session

Participants completed a standardised warm-up consisting of a walk on a treadmill for three minutes at 5–7 km/h, followed by a mobility warm-up of the lower (i.e., glute bridges, leg abduction, and leg adduction) and upper (i.e., external rotation, internal rotation, and Palloff press) extremities. The RT session consisted of exercises performed in the exact order in which the RM session was conducted. Each exercise was performed at 70% of the predicted 1 RM or 6 RM for 3 sets of 10 repetitions with approximately two minutes of rest between each set. Each participant rated their perceived exertion (RPE) after each set using the identical visual analogue scale used during the RM testing session. If the participant’s RPE was ≤7 by the second set, the load was increased by 5%. Conversely, load was decreased by 5% if the full ten repetitions were not completed. The final series of RT exercises consisted of 3 sets of twenty-metre, walking lunges whilst carrying dumbbells and sit-ups whilst clasping an Olympic plate with loads equating to 30% and 10% of the participant’s body mass, respectively. Two minutes of rest were allowed between each set and exercise, and any changes in load during the first RT session were noted, which was then replicated during the second RT session. The RT session concluded with a threeminute warm-down walk on a treadmill at 4–5 km/h.

### 2.8. Statistical Analyses

All data is presented as mean ± standard deviation (SD). According to the Shapiro Wilks test, the majority of the parameters were normally distributed. For parameters that were not normally distributed, the data was transformed using the two-step method [25], which ensured a normal distribution. A two-way (bout [1, 2] × time [Baseline, T24, T48]) repeated measures analysis of variance (ANOVA) was conducted on all measures. For the DOMS measures, given that the baseline measures were not normally distributed as they reported “no soreness” (values ranging from 1–2), only post-exercise time points (T24 and T48) were compared using the two-way repeated measures ANOVA. Differences from baseline to the post-exercise time points in the DOMS measures were determined using a one-sample T-test, with the baseline measure used as the test value. If interaction effects or main effects of time or condition were identified for the repeated measures ANOVA, post hoc analysis was assessed using least square differences to locate differences between the time points. The alpha level was set at <0.05.

## 3. Results

### 3.1. Indirect Muscle Damage Markers

There was no interaction for CK levels (*p* = 0.11; Table 2). However, a significant main effect of time was identified (*p* < 0.01) with significantly higher CK levels at T24 (552.6 ± 279.7 U·L^−1^; *p* < 0.01) and T48 (554.9 ± 310.0 U·L^−1^) than baseline (197.1 ± 99.6 U·L^−1^). There was also a significant main effect of the bout (*p* = 0.002) with significantly higher CK levels for Bout 1 (550.9 ± 242.3 U·L^−1^) than Bout 2 (318.7 ± 164.3 U·L^−1^).

For muscle soreness (Table 2), there were neither interactions nor main time effects for SitSore (*p* = 0.82 and 0.22, respectively), ThighSore (*p* = 0.55 and 0.24, respectively) and HamSore (*p* = 0.79 and 0.06, respectively). However, there was a main effect of bout for all DOMS measures with significantly greater values during Bout 1 than Bout 2 for SitSore (4 ± 2 vs. 3 ± 1; *p* = 0.001), ThighSore (6 ± 3 vs. 3 ± 2; *p* = 0.006) and HamSore (4 ± 2 vs. 2 ± 1; *p* = 0.001). During Bout 1, SitSore and ThighSore scores at T24 and T48 were significantly greater than the baseline score of 1 whilst for HamSore, scores at T24 and T48 were significantly greater than the baseline score of 2 (Table 2). During Bout 2, SitSore scores at T24 and T48 were significantly greater than the baseline score of 1 (Table 2) whilst the ThighSore score at T24 was significantly greater than the baseline score of 1. Results for HamSore were not different from the baseline score of 2 at either follow-up time point.

### 3.2. Anaerobic Performance

There were no interaction effects for CMJ results (*p* = 0.16, Table 3). However, there was a significant main effect of time for CMJ with T24 being significantly lower than baseline (46.8 ± 9.8 vs. 49.3 ± 9.2 cm, *p* < 0.01). No difference in CMJ was found between baseline and T48 (47.95 ± 9.11), and between T24 and T48 (*p* = 0.25). Finally, there was no significant main effect of the bout for CMJ (47.3 ± 9.7 vs. 48.7 ± 9.7 cm, *p* = 0.70).

There was a significant interaction for DJ (*p* = 0.026, Table 3) with significantly lower T24 and T48 measures during Bout 1 than Bout 2. Furthermore, DJ performance at T24 was significantly lower than baseline during Bout 1, although there was no difference between T24 and T48. During Bout 2, no significant difference was found between the different time periods for DJ performance.

There were neither interaction (*p* = 0.50), time main (*p* = 0.17) nor bout (*p* = 0.69) effects for 15 m sprint time (Table 3). For the 10 m sprint times, neither interaction (*p* = 0.10) nor bout (*p* = 0.34) effects were identified (Table 3). However, there was a time main effect for 10 m sprint time (*p* = 0.02) with T48 (2.09 ± 0.24 s) significantly greater than baseline (2.06 ± 0.23 s).

### 3.3. Bowling-Specific Performance

There was no interaction for average bowling speed (*p* = 0.81). However, there was a significant main effect of time for average bowling speed (*p* < 0.01; Table 4), with T24 (95.82 ± 8.12) and T48 (97.04 ± 8.59) being significantly slower than baseline (98.99 ± 8.72), and T24 being significantly slower than T48.

There was also no interaction for run-up time (*p* = 0.38; Table 4). However, there was a significant main effect of time for run-up time (*p* = 0.01) with T24 (2.69 ± 0.60 s) being significantly greater than baseline (2.59 ± 0.60 s). No significant difference was found in run-up time between baseline and T48 (2.67 ± 0.60) or between T24 and T48.

Finally, there was no significant interaction effects for overall bowling accuracy (*p* = 0.88) or RPE (*p* = 0.06; Table 4). Similarly, there was no significant main effect of time (*p* = 0.62; *p* = 0.27; respectively) or bout (*p* = 0.65; *p* = 0.05; respectively) for bowling accuracy and RPE.

## 4. Discussion

This study showed that the elevation in indirect muscle damage markers and impairment in anaerobic performance were greater following the first resistance bout compared with the second resistance session, which partially supported our hypothesis. However, bowling speed was impaired for up to 24 h after both RT bouts, with no differences in bowling accuracy within and between the RT bouts. The results highlight that pace bowlers may need several days of recovery following the first two RT bouts during the commencement of a RT program if incorporating bowling-specific sessions.

The elevated level of indirect muscle damage markers in this study indicated that the first bout of RT likely resulted in EIMD for up to 48 h = post-exercise. These findings are in line with previous studies that reported elevated muscle damage responses in resistance-untrained individuals following similar resistance exercises to that of the current study [16,20]. Elevated levels of CK and increased perception of DOMS are typical signs and symptoms of EIMD [7,8,9] and reflective of muscle fibre degeneration [10]. During periods of EIMD, DOMS results from the disruption of intermediate filaments, leading to the activation of Group III and IV afferents [13]. However, the current study identified that the elevation in DOMS was attenuated following the second RT bout. These findings confirm several studies that reported the RBE phenomenon for DOMS between multi-articular RT bouts [15,16,20]. Interestingly, CK exhibited no interaction effect, possibly due to high inter-individual variability as indicated by the large standard deviation. The inter-individual variability in CK may have been a result of varying competitive levels, age and BMI of the bowlers in the current study [26]. Nonetheless, the main effect of the bout, with larger overall CK values in the first RT bout compared to the second RT, suggests aRBE trend. Whilst still not fully understood, previous research has suggested that EIMD incurred during an initial RT bout elicits structural reorganisation of the intermediate filaments and removes and restores muscle fibres exposed to stress with regenerated fibres, thus preventing further damage in future bouts (i.e., second bout) [19,27]. Subsequent restoration of the intermediate filament may reduce activation of Group III and IV afferents [28] during the second bout, attenuating DOMS. Therefore, the current results provide further evidence that traditional RT can induce a RBE in untrained individuals.

For the anaerobic performance measures, EIMD significantly impaired CMJ, DJ, and 10 m sprint over a 24 and 48 h period. These findings supported previous studies of attenuated linear sprint performance for 24–48 h following a single bout of repeated sprint exercises [21] and resistance exercises [17], with concomitant increases in CK and DOMS. Given that CMJ and DJ were both impaired during periods of EIMD in the current study, it is speculated that the impairment in sprint performance may have been attributed to a compromise in SSC and decreased ability to generate force. It has been suggested that sub-optimal neural recruitment may underpin the compromised use of the stretch-shortening cycle (SSC) and reduced proprioceptive feedback [29]. Interestingly, DJ performance was the only anaerobic performance measure exhibiting a RBE, with no differences identified between the RT bouts for CMJ and sprint performance, suggesting that the level of adaptation across multiple RT bouts is distinct between different types of tasks. Indeed, DJ assesses reactive strength capabilities, with increased eccentric loading and stretch reflex response to optimise the SSC mechanics, and thus, may be more susceptible to performance decrements than the CMJ. In fact, the DJ exhibited a 12% reduction in jump height at T24 in the current study, whilst the difference in jump height for CMJ was 8%, thus allowing participants to better adapt across the RT bouts with the DJ protocol. The lack of RBE trend for sprint performance, when compared to the DJ protocol, could be due to the complexity of the task, affording participants to execute compensatory movements.

The attenuation in bowling-specific performance following the two RT bouts was commensurate with a study by Doma, Leicht, Woods, Harrison, McGuckin and Connor [18], who reported reductions in ball speed for up to 24 h following a RT bout in junior cricket bowlers. These authors suspected that the impairment in bowling performance was a result of mechanical stress, as confirmed by elevation in CK markers. Thus, mechanical stress induced by resistance exercises may have also impaired bowling speed in the current study, as indicated by increases in CK and DOMS, and impairment in CMJ, DJ and 10 m sprint performance. In addition to EIMD markers, there were significant increases in run-up times for up to 24 h post-exercise. Interestingly, a recent meta-analysis revealed that faster linear velocity during the run-up, while maintaining bowling action, resulted in faster ball release speed [2]. Therefore, it is likely that the reduction in run-up speed may in part, have contributed to the decline in average ball speed in the current study. However, there was a disparity in the duration of attenuation between run-up speed (i.e., 24 h post-exercise) and average ball speed (i.e., 48 h post-exercise), suggesting that factors other than run-up speed may influence changes in ball speed during periods of EIMD. Indeed, studies have reported impaired running economy for several hours-to-days following a single bout of traditional resistance exercises with alterations in running gait patterns [30,31]. The authors [30,31] speculated that runners might have exhibited movement compensation in order to limit muscular discomfort experienced during periods of DOMS, thereby increasing the cost of running. Thus, we suspect that the bowlers changed their bowling kinematics as compensation to reduce symptoms of EIMD, leading to decreases in sport-specific performance. However, the comparison between the current study, and those by Doma and Deakin [30,31], should be taken with caution, as running economy is performed at sub-maximal intensities, whilst the protocols in the current study were performed as maximum effort. Further research is necessary to determine whether bowling kinematics are altered meaningfully during periods of EIMD, following traditional resistance exercises.

It was of note that the current study found no change in bowling accuracy despite the observed decline in bowling speed. This was in contrast to work by Doma, Connor, Gahreman, Boullosa, Ahtiainen and Nagata [22] in national-level volleyball players, who reported a reduction in ball speed and improvement in accuracy during a spiking protocol, 24 h following a traditional RT bout. Movement degeneracy [32] may explain the results by Doma, Connor, Gahreman, Boullosa, Ahtiainen and Nagata [22], whereby the volleyball players adapted motor behaviours without compromising function, thus satisfying task constraints (i.e., prioritising spike accuracy over ball speed) [33]. Several authors have highlighted the discrepancy in individual-environment relationships between skilled performers and novices [34,35]. Given that the bowlers in the current study were sub-elite, state-level athletes, they may have lacked the capacity to self-organise under constraints relative to the elite, national-level athletes investigated by Doma, Connor, Gahreman, Boullosa, Ahtiainen and Nagata [22]. Future studies examining sport-specific performance following bouts of RT should incorporate biomechanical parameters to investigate the possibility of movement degeneracy during periods of EIMD.

The current study showed an increase in run-up time and a decline in ball delivery speed following the RT bouts, with no differences identified between the bouts. These findings suggest that the RBE trend was not evident for bowling-specific performance measures, which contradicted our initial hypothesis. This may be due to our bowlers’ limited exposure to EIMD whilst bowling, given they were resistance-untrained. In fact, the current CK values were still greater than 300 U·L^−1^ at 24 and 48 h following the second RT bout in the current study, irrespective of the RBE trend. Such levels of CK have still been reported to impair maximal effort performance, including sprint and agility (Doma et al., 2018; Verma et al., 2016). It is, thus, possible that there was a sufficient level of EIMD present following the second RT bout to impair sprint and bowling-specific performance measures in the current study. Further, Doma, Schumann, Leicht, Heilbronn, Damas and Burt [20] examined CK, DOMS and running time-to-exhaustion measures across three traditional RT bouts. Their results showed that CK and DOMS were minimised, whilst running time-to-exhaustion measures were equally impaired following the first two RT bouts, and only improved following the third RT bout. Interestingly, the current study also showed a reduction in CK and DOMS following the second bout, although no differences were found in the 10-m sprint, run-up time, and bowling speed. Doma, Schumann, Leicht, Heilbronn, Damas and Burt [20] suggested that a greater number of exposures to muscle-damaging exercises may induce further acute adaptations and remodelling of the musculature, also referred to as the repeated-repeated bout effect. It is possible, then, that our bowlers may have exhibited greater improvement in bowling-specific performance measures if they were exposed to a third RT bout. Further research is needed to confirm the repeated-repeated bout effect on bowling-specific performance measures following traditional RT.

Some limitations exist within the current study, which should be noted. First, the majority of the participants recruited were state-level male bowlers. Considering that muscular strength and power and limb length are determinants of bowling-specific performance [2], further research is warranted to determine the impact of EIMD across multiple RT bouts in female bowlers, and those from other skill levels. However, all participants were classified as ‘pace bowler’, which homogenised the sample for some generalisations to be made. Second, as testing took place outdoors, environmental factors such as temperature could not be controlled. However, the reported meteorological data from the local bureau indicated that the ambient temperature during the testing period was stable (23–25 °C). Furthermore, in an attempt to account for this, testing times during the day were kept consistent, and assessments were conducted within the same season. Third, there was a trend for changes with RPE (*p* = 0.06) and bowling accuracy (*p* = 0.05), which suggests that a larger sample size may have exhibited changes at a statistically significant level. Further research with a larger sample size of pace bowlers may confirm the impact of resistance training on perceptual effort and accuracy during pace bowling.

## 5. Conclusions

The repeated-bout effect based upon indirect muscle damage markers and DJ performance was evident for up to 24-h following a second bout of traditional RT in male cricket bowlers. However, no differences were found between the RT bouts for sprint, run-up times, bowling speed and accuracy, only that these measures were impaired following both RT bouts. Together, these results further support a RBE trend for EIMD markers and relatively simple multi-articular movements. Though, it may not be evident in more complex sport-specific tasks, such as pace bowling in cricket. Further research is needed to investigate the influence of EIMD and RBE on athletes’ movement degeneracy and motor skill adaptability, and across a greater number of RT bouts. From a practical standpoint, coaches and athletes seeking to maximise match performance and training adaptations through means of strength and conditioning should be cautious during periods of EIMD. Training sequencing should allow bowling-specific sessions to occur before RT sessions as periods of EIMD have shown to limit performance. Therefore, if bowlers are commencing resistance training for the first time, or are returning from a training hiatus, they may require 48-h of recovery during the first two resistance training bouts.

## Figures and Tables

**Table 1:** Schedule followed for each participant across the study.

	Day 1	Day 2	Day 3	Day 4	Day 5
Week 1	Familiarisation session		RM testing		
Week 2	Tbase		RB	T24	T48
Week 3	Rest week				
Week 4	TBase		RB	T24	T48

RM—repetition maximum; Tbase—baseline testing; RB—resistance training bout; T24—24 h after RB; T48—48 h after RB.

**Table 2:** Means and standard deviation for muscle damage markers before and after the first and second resistance training bouts.

	CK (U/L)	Sit Soreness	Hamstring Soreness	Thigh Soreness
Bout 1				
Baseline	234.5 ± 134.8	1 ± 0	2 ± 2	1 ± 1
T24	675.6 ± 299.7	4 ± 1 ^b^	5 ± 2 ^d^	6 ± 3 ^b^
T48	742.5 ± 512.0	4 ± 3 ^b^	4 ± 2 ^c^	5 ± 3 ^b^
Bout 2				
Baseline	159.6 ± 78.5	1 ± 1	2 ± 1	1 ± 1
T24	429.5 ± 299.1	3 ± 2 ^b^	3 ± 2	3 ± 3 ^a^
T48	367.2 ± 205.1	2 ± 1 ^a^	2 ± 2	3 ± 2

T24 and T48—24 and 48 h after the resistance training bout. ^a^ *p* < 0.05; ^b^ *p* < 0.01 greater than 1 ^c^ *p* < 0.05; ^d^ *p* < 0.01 greater than 2.

**Table 3:** Means and SD for anaerobic performance measures before and after bout 1 and bout 2 of resistance training.

	CMJ (cm)	DJ (cm)	15 m Sprint	10 m Sprint
Bout 1				
Baseline	51.5 ± 10.6	47.1 ± 11.5	2.57 ± 0.22	2.04 ± 0.22
T24	47.5 ± 10.5	41.9 ± 8.8 *	2.67 ± 0.20	2.14 ± 0.25
T48	49.3 ± 9.0	44.1 ± 10.6	2.65 ± 0.25	2.12 ± 0.28
Bout 2				
Baseline	51.8 ± 10.8	47.4 ± 9.6	2.61 ± 0.22	2.07 ± 0.24
T24	50.6 ± 10.7	46.9 ± 8.0 ^a^	2.64 ± 0.22	2.10 ± 0.23
T48	50.6 ± 9.9	48.0 ± 8.2 ^b^	2.61 ± 0.24	2.06 ± 0.20

CMJ—countermovement jump; DJ—drop jump; T24 and T48—24 and 48 h after the resistance training bout. * *p* < 0.05 compared with baseline; ^a^ *p* < 0.05 compared with Bout 1 T24; ^b^ *p* < 0.05 compared with Bout 1 T48.

**Table 4:** Means and SD for bowling-specific performance measures before and after Bout 1 and Bout 2 of resistance training.

	Ball Speed (km/h)	Accuracy (%)	Run-Up Time (s)	RPE
Bout 1				
Baseline	98.4 ± 8.9	31.7 ± 9.5	2.63 ± 0.61	12 ± 2
T24	95.1 ± 8.3	33.7 ± 7.9	2.70 ± 0.59	14 ± 3
T48	96.7 ± 9.1	29.4 ± 8.8	2.68 ± 0.60	13 ± 3
Bout 2				
Baseline	99.62 ± 8.8	30.7 ± 13.9	2.56 ± 0.59	12 ± 3
T24	96.57 ± 8.3	32.0 ± 10.2	2.67 ± 0.62	12 ± 3
T48	97.37 ± 8.4	29.7 ± 10.7	2.65 ± 0.64	12 ± 3

T24 and T48—24 and 48 h after the resistance training bout.

## Data Availability

The data presented in this study are available on request from the corresponding author.

## Abbreviations

ANOVA	analysis of variance
Tbase	Baseline
T24	24 h post-resistance training
T48	48 h post-resistance training
RT	Resistance training
EIMD	Exercise-induced muscle damage
CK	Creatine kinase
RBE	Repeated bout effect
DJ	Drop jump
RM	Repetition maximum
DOMS	Delayed-onset muscle soreness
CMJ	Counter-movement jump
RPE	Rating of perceived exertion
SD	Standard deviation
SSC	Stretch-shortening cycle
